# Fruit and Vegetable Consumption and Incident Prefrailty and Frailty in Community-Dwelling Older People: The English Longitudinal Study of Ageing

**DOI:** 10.3390/nu12123882

**Published:** 2020-12-18

**Authors:** Gotaro Kojima, Steve Iliffe, Stephen Jivraj, Kate Walters

**Affiliations:** 1 Department of Primary Care and Population Health, University College London, London NW3 2PF, UK; s.iliffe@ucl.ac.uk (S.I.); k.walters@ucl.ac.uk (K.W.); 2 Department of Research, Dr. AGA Clinic, Tokyo 105-0004, Japan; 3 Department of Epidemiology and Public Health, University College London, London WC1E 7HB, UK; stephen.jivraj@ucl.ac.uk

**Keywords:** frailty, fruit, vegetables, diet, nutrition, community-dwelling older people

## Abstract

Background: There is limited evidence in the literature regarding associations between fruit and vegetable consumption and risk of frailty. Objective: To examine associations between fruit and vegetable consumption and risk of incident frailty and incident prefrailty/frailty. Design: A prospective panel study. Setting and Subjects: 2634 non-frail community-dwelling men and women aged 60 years or older from the English Longitudinal Study of Ageing (ELSA). Methods: Fruit and vegetable consumption/day was measured using a self-completion questionnaire at baseline. Frailty status was measured at baseline and follow-up was based on modified frailty phenotype criteria. Four-year incident frailty was examined among 2634 robust or prefrail participants, and incident prefrailty/frailty was measured among 1577 robust participants. Results: Multivariable logistic regression models adjusted for age, gender, and other confounders showed that fruit and vegetable consumption was not associated with incident frailty risks among robust or prefrail participants. However, robust participants consuming 5–7.5 portions of 80 g per day (odds ratio (OR) = 0.56, 95% confidence interval (CI) = 0.37–0.85, *p* < 0.01) and 7.5–10 portions per day (OR = 0.46, 95%CI = 0.27–0.77, *p* < 0.01) had significantly lower risk of incident prefrailty/frailty compared with those consuming 0–2.5 portions/day, whereas those consuming 10 or more portions/day did not (OR = 1.10, 95%CI = 0.54–2.26, *p* = 0.79). Analysis repeated with fruit and vegetable separately showed overall similar results. Conclusions: Robust older adults without frailty who eat current U.K. government recommendations for fruit and vegetable consumption (5–10 portions/day) had significantly reduced risks of incident prefrailty/frailty compared with those who only eat small amount (0–2.5 portions/day). Older people can be advised that eating sufficient amounts of fruit and vegetable may be beneficial for frailty prevention.

## 1. Introduction

Frailty has been described as a geriatric condition characterised by inability to respond to outer and inner stressors to sustain homeostasis due to age-related decreased multisystem reserves resulting from accumulated health deficits [1]. In the most frequently used criteria, which were advocated by Fried et al. using the Cardiovascular Health Study (CHS) cohort, frailty was defined as having three or more of five physical components: unintentional weight loss, exhaustion, weakness, slow gait speed, and low physical activity [2]. Frail older people are predisposed to various negative health outcomes, such as disabilities, emergency department visits, and premature death [3]. Although advanced age is a strong risk factor of frailty and in general it becomes more prevalent as people age, frailty is a dynamic process that can both improve or progress [4,5]. Given these detrimental effects on patients, families, healthcare systems, and societies and its potential reversibility, frailty has been considered as an urgent public health priority [6,7].

More recently, there has been growing evidence on relationships between frailty and diet [8]. Healthy dietary patterns (e.g., Mediterranean diet) and some nutrients (e.g., protein) appear to be related to lower frailty risks [9,10,11,12,13]. Diet is one of the most important factors for maintaining good health, and poor dietary patterns may lead to obesity, malnutrition, and chronic diseases, such as hypertension, diabetes, hyperlipidemia, cardiovascular diseases, and cancers [14,15]. Among various dietary components, fruit and vegetables have been recognised as key components of healthy diet due to their high concentrations of nutrients, including dietary fibre, minerals, vitamins, and antioxidants [16]. In 2003, the World Health Organization and the Food and Agriculture Organization of the United Nations launched a joint initiative to recommend that people should eat at least 400 g of fruit and vegetables per day to promote good health [17]. In the United Kingdom, the United States, France, Germany, and many other countries, consumption of at least five portions of fruit and vegetables (approximately 80 g/portion) per day has been recommended by “5 A Day” campaigns [18]. In some countries, even higher amounts than 400 g/day have been recommended. A recent dose-response meta-analysis revealed that an even higher amounts of fruit and vegetable consumption than 400 g/day are associated with health benefits among adult populations aged 16 or older [19]. Risks of cancer reduced up to 600 g (7.5 portions) per day, and risks of coronary heart disease, stroke, cardiovascular diseases, and all-cause mortality reduced up to 800 g (10 portions) per day [19].

However, there is limited evidence in the literature, with mixed results, regarding associations between fruit and vegetable consumption and frailty [20]. A recent systematic review found several related papers, but most of them examined the association as a supplementary analysis or were considered to have suboptimal methodological quality [20]. Therefore, no clear conclusion was able to be drawn by the review [20]. In contrast, a clear protective effect is seen for adhering to a Mediterranean diet and incident frailty, with those with highest adherence having less than half the risk of frailty compared with the lowest adherence [11]. We do not know, however, whether it is the high fruit and vegetable content or other elements of this diet that may confer benefit in preventing frailty onset. In light of the scarce and conflicting results, we aimed to investigate the association between fruit and vegetable consumption and incident frailty among community-dwelling older people in England. Our hypothesis was that higher fruit and vegetable consumption was associated with lower risk of incident frailty.

## 2. Methods

### 2.1. Study Setting and Population

This study used a sample of men and women aged 60 or older from the English Longitudinal Study of Ageing (ELSA) and followed the STROBE (Strengthening the Reporting of Observational Studies in Epidemiology) guidelines [21]. This is an ongoing multi-centre longitudinal panel study of a nationally representative community-dwelling population who are 50 years and older in England [22]. ELSA was launched in 2002 with a specific focus on investigating and understanding the ageing process, covering a broad range of topics, such as physical and mental health, cognitive function, social and economic conditions, well-being, and relationships among these factors in older people [22]. Participants were initially recruited at wave 1 in 2002 from households of participants of the Health Survey for England (HSE) in 1998, 1999, and 2001 [23]. HSE is an annual cross-sectional survey to monitor the health of the general population in England [24]. The panel of ELSA has been followed every two years. Participants who had the main interview at every second wave starting from wave 2 in 2004 (i.e., waves 2, 4, 6, and 8) were invited to have a nurse visit, where physical function and anthropometry information were measured and a blood sample was drawn [23]. New sample groups were added from HSE at waves 3, 4, and 6 in order to ensure the representativeness of the ELSA [22]. For the current study, data at waves 4 and 6 were used. Ethical approval was obtained from the National Research and Ethics Committee (MREC/01/2/91). The ELSA study was conducted in accordance with the Declaration of Helsinki [25]. Details of the ELSA study design, sample, and data collection are available at the ELSA project website (https://www.elsa-project.ac.uk/).

Of 9986 core members who had an interview at wave 4 in 2008, 2895 participants aged 50–59 years were removed as they were not eligible for the nurse visit and therefore did not have their gait speed, body height, and weight measured. In addition, 2184 and 540 participants were excluded due to missing data for frailty and fruit and vegetable consumption, respectively. Among 4267 participants for whom data on frailty and fruit and vegetable consumption were available at baseline, 611 did not return and 534 did not have complete data for frailty at follow-up (wave 6 in 2012), leaving 3122 participants. Among those, 196 frail participants and 292 participants who had implausibly high values of fruit and vegetable consumption were removed, which left 2634 non-frail participants (1577 robust and 1057 prefrail) that were used for the current study.

### 2.2. Outcome Variable: Incident Frailty

Frailty was measured at wave 4 in 2008 (baseline) and wave 6 in 2012 (follow-up) based on the CHS criteria [2] with slight modifications on weight loss, exhaustion, and low physical activity criteria because of data availability [26]. The five criteria are as follows: (1) Unintentional weight loss was defined as a body mass index <18.5 kg/m^2^ or decreasing body weight 5% or more between waves 2 and 4 for the core members participating from the beginning of ELSA (*n* = 2132) and between HSE in 2006 and wave 4 for the new cohort participants who joined at wave 4 (*n* = 502). (2) Exhaustion was defined as answering yes to “(Much of the time during the past week), you felt that everything you did was an effort?” or “(Much of the time during the past week), you could not get going?”, which are two questions from the Center for Epidemiological Studies-Depression (CES-D) scale [27]. (3) Weakness was defined as being in the gender- and body mass index-stratified lowest 20% of handgrip strength using Smedley hand-held dynamometer (Stoelting Co, Wood Dale, IL, USA). (4) Slow gait speed was defined as being in the gender- and height-stratified lowest 20% of average gait speed of two trials of 8-foot walking at a usual pace. Participants who were in a wheelchair, bed-bound, or unable to walk without assistance were considered to have the slowest gait speed. (5) Low physical activity was defined as being in the lowest two physical activity categories out of four, based on usual exercise in terms of intensity (vigorous, moderate, or mild) and frequency (more than once per week, once per week, one to three times per month, or hardly ever or never). Those who had zero, one to two, and three to five components were defined as robust, prefrail, and frail, respectively [2]. Frailty status at wave 6 was defined as described above except for the unintentional weight loss, which was defined as a body mass index <18.5 kg/m^2^ or decreasing body weight 5% or more between waves 4 and 6.

### 2.3. Explanatory Variables

#### Fruit and Vegetable Consumption

Information on consumption of fruit and vegetables on the day prior to the interview was solicited by self-completion questionnaires (Supplementary Figure S1), [22] which consists of four questions for vegetables and nine questions for fruit and was derived from the Welsh Health Survey [28]. The amount of consumed fruit and vegetables were converted into portions (1 portion = 80 g for both vegetables and fruit) in accordance with the Welsh Health Survey methodology and the “5 A Day” campaign portion size from the National Health Service (NHS) [29]. Conversion rates are shown in Supplementary Figure S1. Implausibly high values were defined as values more than mean value plus 3 standard deviations for each fruit and vegetable consumption, and those with at least one implausibly high value were excluded from analysis (*n* = 292). The total portion of fruit and vegetable consumption was calculated as a sum of fruit and vegetable consumption and was divided into five groups (≥0–2.5, ≥2.5–5, ≥5–7.5, ≥7.5–10, and >10 portions of 80 g per day). The cut-points were chosen based on the “5 A Day” campaign and the recent findings on beneficial effects of fruit and vegetables with a higher amount than 5 portions (7.5 and 10 portions) [19,29]. In analyses of consumption of fruit and vegetables separately, cut-points were 1.5, 3, 4.5, and 6 for fruit and 1, 2, 3, and 4 for vegetables. These cut-points were decided proportional to an approximate ratio (3:2) of median values of fruit and vegetable consumption in this cohort.

### 2.4. Confounders

Confounders that were considered in this study were age, gender, smoking, alcohol use, wealth, education, living alone, cognition, depressed mood, diabetes, and hyperlipidemia. Age was grouped into five-year cohorts: (1) 60–64 years old, (2) 65–69 years old, (3) 70–74 years old, (4) 75–79 years old, and (5) 80 years and older. Smoking status was categorised into (1) current smokers and (2) non-smokers. Alcohol use was categorised based on frequency into (1) none, (2) once a year to twice a month, (3) once a week to four days a week, and (4) five days a week or more. Wealth was calculated as a sum of savings, investments, physical wealth, and housing wealth deducting financial debt and mortgage debt and then grouped into quintiles. Education was divided into higher education (national vocational qualification (NVQ) level 4, level 5, degree, or equivalent), intermediate education (NVQ level 1/Certificate of Secondary Education equivalent, level 2/General Certificate of Education (GCE) O level equivalent, level 3/GCE A level equivalent, higher education below degree, or foreign/other qualification), and no qualification. Cognition was measured using a total score of four tests: animal naming task, letter cancellation task, and immediate and delayed recall tasks, with a higher score suggestive of better cognitive function [23]. Depressed mood was measured using a depressive mood subscale, [30] comprising five items from the CES-D scale (not including the two items used to define frailty). This depressive mood score ranged from 0 to 5, with a higher score indicative of more depressed mood. Diabetes was confirmed by asking participants if they were ever told by a doctor that they had or had previously had diabetes or high blood sugar or they were taking diabetic medication or insulin. Hyperlipidemia was confirmed by participants if they were ever told by a doctor that they had or had previously had high cholesterol.

### 2.5. Statistical Analysis

Multivariable logistic regression models were used to calculate odds ratios (ORs) of incident frailty among the non-frail (i.e., robust and prefrail) and incident prefrailty/frailty among the robust by total fruit and vegetable consumption, adjusting for age, gender, smoking, alcohol use, wealth, education, living alone, cognition, depressed mood, diabetes, and hyperlipidemia. The reference group was the lowest total consumption group (≥0–2.5 portions/day). The same analyses were repeated for fruit and vegetables separately.

Three supplementary analyses were performed. First, the fully adjusted models were repeated using multiple imputation by chained equations for missing data of the covariates that were used for adjustment. We assumed that the data were missing at random and the probability of missingness was not dependent on unobserved data but on observed data. In the other supplementary analyses, the implausibly high values were treated as 0 (worst case scenario) or mean plus 3 standard deviations, respectively, and the fully adjusted models were repeated.

A longitudinal weighting calculating the response bias at each wave and between waves was used for all analyses. All statistical analyses were conducted using StataSE 14 (StataCorp LP, College Station, TX, USA) and were based on 2-sided significance of *p* value < 0.05.

## 3. Results

Table 1 shows baseline characteristics of 2634 participants into five groups based on total fruit and vegetable consumption. The amount of total fruit and vegetable consumption ranged from 0 to 17 portions per day, and the distribution was skewed to the right. The number of participants was 323, 913, 971, 329, and 98 for the first (the lowest), second, third, fourth, and fifth (the highest) groups, respectively. Although there is no clear dose-response relationship between baseline characteristics and fruit and vegetable consumption, the lower consumption groups were more likely than the other groups to be frailer, men, current smokers, non-drinkers, living alone, and diabetic and were more likely to have higher body mass index, lower wealth, less education, and poorer cognitive function.

Multivariable logistic regression models calculated ORs of incident frailty for daily consumption of total fruit and vegetables as well as fruit and vegetables separately in 2634 non-frail (robust and prefrail) participants are depicted in Table 2. Those consuming 5–7.5 portions of fruit and vegetables had a significantly lower risk than those consuming 0–2.5 portions in Model 1 (OR = 0.58, 95% confidence interval (CI) = 0.35–0.98, *p* = 0.04). These associations became nonsignificant in the fully adjusted Model 3 (OR = 1.03, 95% CI = 0.58–1.84, *p* = 0.91). Those consuming 3–4.5 and 4.5–6 portions of fruit per day had significantly lower risks than those consuming 0–1.5 portions per day only in the age- and gender-adjusted model (OR = 0.56, 95% CI = 0.34–0.92, *p* = 0.02 for 3–4.5 portions/day; OR = 0.56, 95% CI = 0.31–0.99, *p* = 0.05 for 4.5–6 portions/day) but not in the fully adjusted model (OR = 0.93, 95% CI = 0.53–1.65, *p* = 0.81 for 3–4.5 portions/day; OR = 1.05, 95% CI = 0.55–2.01, *p* = 0.87 for 4.5–6 portions/day). There was no significant association with vegetable consumption in any models.

Table 3 shows the results of multivariable logistic regression models for incident prefrailty/frailty among 1577 robust participants. For total fruit and vegetable consumption, those who consumed 5–7.5 and 7.5–10 portions per day had significantly lower risks of incident prefrailty/frailty compared with those who consumed 0–2.5 portions per day in all models (Model 3: OR = 0.56, 95% CI = 0.37–0.85, *p* < 0.01 for 4–7.5 portions/day; OR = 0.46, 95% CI = 0.27–0.77, *p* < 0.01 for 7.5–10 portions/day). For fruit consumption, 3–4.5 and 4.5–6 portions per day were significantly associated with lower risks of incident prefrailty/frailty compared with 0–1.5 portions per day in Model 1, which remained significant in Models 2 and 3 (Model 3: OR = 0.58, 95% CI = 0.40–0.84, *p* < 0.01 for 3–4.5 portions/day; OR = 0.52, 95% CI = 0.33–0.81, *p* < 0.01 for 4.5–6 portions/day). For vegetable consumption, all groups consuming 1 or more portions of vegetables per day had significantly lower risks of incident prefrailty/frailty compared with those consuming 0–1 portion. In Model 3, 1–2, 2–3, and 4–9 portions of vegetables per day remained significantly associated with lower risks (Model 3: OR = 0.57, 95% CI = 0.38–0.86, *p* < 0.01 for 1–2 portions/day; OR = 0.64, 95% CI = 0.43–0.96, *p* = 0.03 for 2–3 portions/day; OR = 0.53, 95% CI = 0.30–0.93, *p* = 0.03 for 4 portions or more/day).

Three supplementary analyses were performed. Model 3 was separately repeated (1) imputing missing data of covariates used for adjustments using multiple imputation by chained equations, (2) treating implausibly high values of fruit and vegetable consumption as 0, and (3) treating implausibly high values of fruit and vegetable consumption as mean plus 3 standard deviations. The results were essentially not different from those of the main analyses.

## 4. Discussion

In this study of English community-dwelling older men and women aged 60 years or over, fruit and vegetable consumption did not have significant effects on risk of incident frailty among non-frail participants (i.e., robust and prefrail). However, among robust participants, moderate fruit and vegetable consumption (5–10 portions/day) was significantly associated with reduced risks of newly developing prefrailty or frailty, compared with the lowest consumption, whereas the highest consumption (>10 portions/day) was not.

There are three studies with the main focus on the association between fruit and vegetable consumption and incident frailty [31,32,33]. Fruit and vegetables were measured by quantity [31,32] or frequency [33].

A study used 2926 non-frail (robust or prefrail) community-dwelling older people from three cohorts (two from France and one from Spain) and showed that consumption of fruit, vegetables, and fruit and vegetables combined were all inversely associated with incident frailty over 2.5 years in a dose-response manner [31]. Differences between countries, such as price and availability of fruit and vegetables or economic status of older people, may explain some of the differences observed.

The other study measuring fruit and vegetables by quantity examined risk of incident frailty in 78,366 non-frail women aged 60 years or older from the Nurses’ Health Study [32]. Those who consumed seven or more servings of fruit and vegetables were significantly less likely to develop frailty than those who consumed less than three servings over 20 years (hazard ratio = 0.92, 95% CI = 0.85–0.99) in a dose response manner (*p* for trend = 0.03) [32]. Although these studies demonstrated significant associations with incident frailty, in our study significant associations were observed not with incident frailty but only with incident prefrailty/frailty. Their study also showed a dose-response relationship between fruit and vegetable consumption and frailty risk, but our study showed a U-shaped association where the lowest and highest fruit and vegetable consumption had higher risks than moderate consumption.

The last study using the Whitehall II study cohort measured fruit and vegetables by frequency and showed that those eating fruit and vegetables at least twice a day had a significantly lower risk of developing frailty during 20-year follow-up (hazard ratio = 0.70, 95% CI = 0.53–0.92, *p* = 0.01) compared with those eating less than once a day [33].

The current study showed that incident prefrailty/frailty risk of the highest fruit and vegetable consumption was not different from that of the lowest consumption group, whereas the moderate consumption significantly decreased prefrailty/frailty risks. The reasons for these findings are not clear, but may be attributable to a possible ceiling effect of health benefits from fruit and vegetable consumption as shown in the dose-response meta-analysis study that more than 10 portions of fruit and vegetable consumption failed to show additional benefits against mortality and cardiovascular diseases [19]. Another possible cause is that a very high amount of fruit and vegetable consumption may hinder consuming sufficient calories or other important nutrients, such as protein and result in unbalanced, poor-quality diet. Although containing multiple micro-nutrients, fruit and vegetables are not calorie dense or protein-rich foods and low intake of calories or protein is known to be associated with increased risks of frailty [9,12].

There are several potential mechanisms by which fruit and vegetable consumption may possibly prevent the development of frailty. A recent systematic review and meta-analysis revealed that frailty was associated with increased oxidative stress markers and possibly with decreased antioxidant parameters [34]. Oxidative stress can increase reactive oxygen species production and damage DNA, lipids, and proteins and cause mitochondrial dysfunction and apoptosis [10], possibly leading to frailty. Fruit and vegetables are natural sources of multiple beneficial nutrients, including vitamin C, vitamin E, carotenoids, and selenium. These nutrients can act as antioxidants and decrease reactive oxygen species [10]. Inflammation has been considered to play a role in the development of frailty [35,36]. Frail and prefrail older people have significantly higher levels of inflammatory markers, such as C-reactive protein, interleukin-6, white blood cells, and fibrinogen [37]. Some compounds of fruit and vegetables have been shown to have anti-inflammatory effects [38,39,40], through which fruit and vegetables may decrease frailty risk. Another possibility is that high fruit and vegetable consumption represents a surrogate marker of high-quality dietary patterns or healthy lifestyles. Those who are more health conscious may choose a healthier lifestyle, including consuming more fruit and vegetables. Evidence has shown that specific dietary patterns, such as following the Mediterranean diet [41] and adhering to high dietary quality [9] are significantly associated with lower risk of frailty. Further research should explore the main fruit and vegetable sub-types and their association with frailty, alongside which compounds are more important in this association (e.g., fibre, micronutrients) to understand these relationships better.

The findings should be interpreted with caution. One of the limitations regarding data on fruit and vegetable consumption used in the present study is that the information of fruit and vegetable consumption on the day prior to the interview was obtained by a self-completed questionnaire, which may be subject to recall bias and affect the generalisability of the findings. Limited data are available on the validity and reliability of this measure; however, it has been widely used in surveys in the United Kingdom [28], and more robust instruments are often limited by their ease of use in population surveys. Second, in this study, the frailty phenotype criteria were used with some slight modifications due to data availability. Such modifications have been common in other frailty studies, but they may have a significant influence on the results [42]. Third, our analytic sample (*n* = 2634) was approximately one-third of the core sample aged 60 or older at baseline (*n* = 7091), and two-thirds of the sample was excluded due to missing data or attrition. Those who had frailty information at baseline wave 4 but were excluded due to missing data for fruit and vegetable consumption at baseline or frailty at follow-up wave 6 (*n* = 1328) were significantly more likely to be prefrail rather than robust, older, current smokers, and non-drinkers and more likely to have lower education, lower wealth, and more depressed mood and live alone. These differences may generate a selection bias towards the null hypothesis. To account for attrition bias, non-response weighting was used in the analyses [22]. Repeating the analyses with missing data of covariates imputed did not significantly change the results. Fourth, although total kilocalories and protein intake were considered to be important confounders, the data were not available. Fifth, only frequency but not amount of alcohol was available for the analysis. Sixth, the information of diabetes and hyperlipidemia was reported by the participants but was not based on physicians’ diagnosis. Lastly, ELSA cohort consisted of mainly white British people in England, and our findings may not be generalisable to other populations.

This study’s strengths include the prospective study design with a large number of older men and women. The analyses were performed based on total consumption of fruit and vegetables as well as fruit and vegetables separately and were controlled for a wide range of potential confounders. In addition, supplementary analyses were conducted to address missing values and potential outliers with implausibly high values, both of which did not change the results significantly.

In conclusion, moderate consumption of fruit and vegetable was significantly associated with lower risks of developing prefrailty/frailty among robust older people in England. Sufficient amounts of fruit and vegetables may be an important lifestyle factor against frailty among older people. In light of scarce information available on this topic in the literature, more high-quality prospective longitudinal cohort studies are warranted, using valid and reliable measures of fruit and vegetable consumption and frailty.

## Figures and Tables

**Table 1:** Baseline characteristics and incident prefrailty and frailty among 2634 non-frail (prefrail or robust) community-dwelling older people in England by daily total fruit and vegetable consumption.

Variable *	Entire Cohort *n* = 2634	0–<2.5 Portions *n* = 323	2.5–<5 Portions *n* = 913	5–<7.5 Portions *n* = 971	7.5–<10 Portions *n* = 329	≥10–17 Portions *n* = 98
Frailty status (missing = 0)						
Robust	1577 (59.9%)	174 (53.9)	556 (60.9)	581 (59.8)	212 (64.4)	54 (55.1)
Pre-frail	1057 (40.1%)	149 (46.1)	357 (39.1)	390 (40.2)	117 (35.6)	44 (44.9)
Incident frailty rate robust & prefrail (case/total)	0.072 (189/2634)	0.096 (31/323)	0.079 (72/913)	0.062 (60/971)	0.06 1(20/329)	0.06 1(6/98)
Incident prefrailty/frailty rate robust only (case/total)	0.317 (500/1577)	0.39 1(68/174)	0.336 (187/556)	0.29 1(169/581)	0.255 (54/212)	0.407 (22/54)
Age group (missing = 0)						
60–64	941 (35.7%)	129 (39.9%)	345 (37.8%)	319 (32.9%)	121 (36.8%)	27 (27.6%)
65–69	645 (24.5%)	82 (25.4%)	211 (23.1%)	236 (24.3%)	89 (27.1%)	27 (27.6%)
70–74	606 (23.1%)	60 (18.6%)	206 (22.6%)	248 (25.5%)	72 (21.9%)	20 (20.4%)
75–79	275 (10.4%)	33 (10.2%)	95 (10.4%)	110 (11.3%)	24 (7.3%)	13 (13.3%)
80+	167 (6.3%)	19 (5.9%)	56 (6.1%)	58 (6.0%)	23 (7.0%)	11 (11.2%)
Female	1471 (55.9%)	157 (48.6%)	490 (53.7%)	585 (60.3%)	191 (58.1%)	48 (49.0%)
BMI (missing = 0)	28.1 ± 4.7	28.6 ± 4.7	28.0 ± 4.7	28.0 ± 4.7	28.0 ± 4.8	27.9 ± 4.4
Smoking (missing = 18)						
Current smoker	218 (8.3%)	56 (17.5%)	93 (10.3%)	53 (5.5%)	8 (2.5%)	8 (8.3%)
Non-current smoker	2398 (91.7%)	265 (82.6%)	814 (89.8%)	913 (94.5%)	317 (97.5%)	89 (91.8%)
Alcohol (missing = 19)						
None	205 (7.8%)	39 (12.2%)	70 (7.7%)	73 (7.6%)	17 (5.2%)	6 (6.2%)
1/year-2/month	694 (26.5%)	82 (25.6%)	247 (27.3%)	248 (25.7%)	90 (27.5%)	27 (27.8%)
1/week-4/week	1,043 (39.9%)	117 (36.6%)	352 (38.8%)	386 (40.0%)	143 (43.7%)	45 (46.4%)
5/week-daily	673 (25.7%)	82 (25.6%)	238 (26.2%)	257 (26.7%)	77 (23.6%)	19 (19.6%)
Wealth quintile (missing = 48)						
Richest	702 (27.2%)	52 (16.5%)	219 (24.4%)	295 (31.0%)	109 (33.8%)	27 (28.1%)
2nd	620 (24.0%)	52 (16.5%)	233 (26.0%)	246 (25.8%)	77 (23.8%)	12 (12.5%)
3rd	568 (22.0%)	73 (23.1%)	207 (23.1%)	194 (20.4%)	71 (22.0%)	23 (24.0%)
4th	423 (16.4%)	80 (25.3%)	143 (15.9%)	141 (14.8%)	38 (11.8%)	21 (21.9%)
Poorest	273 (10.6%)	59 (18.7%)	96 (10.7%)	77 (8.1%)	28 (8.7%)	13 (13.5%)
Education (missing = 0)						
Higher education	468 (17.8%)	30 (9.3%)	160 (17.5%)	183 (18.9%)	75 (22.8%)	20 (20.4%)
Intermediate	1524 (57.9%)	169 (52.3%)	539 (59.0%)	578 (59.5%)	182 (55.3%)	56 (57.1%)
No qualification	642 (24.4%)	124 (38.4%)	214 (23.4%)	210 (21.6%)	72 (21.9%)	22 (22.5%)
Living alone (missing = 0)	610 (23.2%)	84 (26.1%)	183 (20.0%)	235 (24.2%)	83 (25.2%)	25 (25.5%)
Cognitive function (missing = 183)	51.8 ± 12.3	50.1 ± 10.6	51.6 ± 10.4	52.2 ± 10.0	52.8 ± 11.1	50.9 ± 12.3
Depressive mood (missing = 11)	0.5 ± 1.0	0.6 ± 1.2	0.4 ± 0.9	0.5 ± 1.0	0.5 ± 1.1	0.6 ± 1.3
Diabetes (missing = 0)	239 (9.1%)	40 (12.4%)	87 (9.5%)	75 (7.7%)	26 (7.9%)	11 (11.2%)
Hyperlipidemia (missing = 0)	1077 (40.9%)	132 (40.9%)	387 (42.4%)	401 (41.3%)	119 (36.2%)	38 (38.8%)

* Mean ± standard deviation or *n* (%). BMI: body mass index.

**Table 2:** Associations between daily fruit and vegetable consumption and incident frailty risk over 4 years among 2634 non-frail community-dwelling older people in England.

Model	Model 1		Model 2		Model 3	
Variable	Odds Ratio (95% CI)	*p*-value	Odds Ratio (95% CI)	*p*-value	Odds Ratio (95% CI)	*p*-value
Fruit						
0–<1.5 portions	ref		ref		ref	
1.5–<3 portions	0.71 (0.44–1.15)	0.16	0.94 (0.57–1.54)	0.79	1.07 (0.63–1.82)	0.79
3–<4.5 portions	0.56 (0.34–0.92)	0.02	0.76 (0.44–1.30)	0.31	0.93 (0.53–1.65)	0.81
4.5–<6 portions	0.56 (0.31–0.99)	0.05	0.75 (0.41–1.39)	0.36	1.05 (0.55–2.01)	0.87
6–11 portions	0.92 (0.47–1.80)	0.80	1.40 (0.68–2.89)	0.36	1.98 (0.93–4.22)	0.08
Vegetable						
0–<1 portion	ref		ref		ref	
1–<2 portions	0.79 (0.47–1.32)	0.37	1.09 (0.62–1.91)	0.77	1.24 (0.67–2.28)	0.49
2–<3 portions	0.97 (0.58–1.63)	0.92	1.33 (0.76–2.34)	0.32	1.54 (0.84–2.84)	0.16
3–<4 portions	0.65 (0.34–1.24)	0.19	0.93 (0.46–1.88)	0.84	1.05 (0.49–2.22)	0.90
4–9 portions	0.46 (0.20–1.04)	0.06	0.57 (0.24–1.36)	0.20	0.53 (0.20–1.36)	0.19
Total						
0–<2.5 portions	ref		ref		ref	
2.5–<5 portions	0.77 (0.46–1.28)	0.32	1.06 (0.62–1.80)	0.83	1.22 (0.68–2.17)	0.50
5–<7.5 portions	0.58 (0.35–0.98)	0.04	0.87 (0.50–1.49)	0.61	1.03 (0.58–1.84)	0.91
7.5–<10 portions	0.56 (0.28–1.12)	0.10	0.91 (0.43–1.93)	0.82	1.19 (0.54–2.65)	0.67
10–17 portions	0.44 (0.17–1.17)	0.10	0.51 (0.18–1.41)	0.20	0.60 (0.21–1.77)	0.36

Model 1: Adjusted for age and gender. Model 2: Adjusted for age, gender, smoking, alcohol, wealth, education, and living alone. Model 3: Adjusted for age, gender, smoking, alcohol, wealth, education, living alone, cognition, depressed mood, diabetes, and hyperlipidaemia. CI: confidence interval, ref: reference.

**Table 3:** Associations between daily fruit and vegetable consumption and incident prefrailty/frailty risk over 4 years among 1577 robust community-dwelling older people in England.

	Model 1		Model 2		Model 3	
Variable	Odds Ratio (95% CI)	*p*-Value	Odds Ratio (95% CI)	*p*-Value	Odds Ratio (95% CI)	*p*-Value
Total						
0–<2.5 portions	ref		ref		ref	
2.5–<5 portions	0.76 (0.52–1.11)	0.20	0.90 (0.61–1.33)	0.59	0.77 (0.52–1.15)	0.21
5–<7.5 portions	0.54 (0.37–0.79)	<0.01	0.66 (0.44–0.99)	0.04	0.56 (0.37–0.85)	<0.01
7.5–<10 portions	0.43 (0.27–0.68)	<0.001	0.55 (0.34–0.91)	0.02	0.46 (0.27–0.77)	<0.01
10–17 portions	0.93 (0.47–1.84)	0.84	1.16 (0.59–2.29)	0.66	1.10 (0.54–2.26)	0.79
Fruit						
0–<1.5 portions	ref		ref		ref	
1.5–<3 portions	0.82 (0.58–1.18)	0.28	0.96 (0.67–1.39)	0.84	0.86 (0.59–1.26)	0.45
3–<4.5 portions	0.55 (0.39–0.78)	0.001	0.66 (0.46–0.95)	0.02	0.58 (0.40–0.84)	<0.01
4.5–<6 portions	0.56 (0.37–0.83)	<0.01	0.64 (0.42–0.98)	0.04	0.52 (0.33–0.81)	<0.01
6–11 portions	0.82 (0.48–1.39)	0.46	1.12 (0.66–1.90)	0.67	1.17(0.67–2.02)	0.58
Vegetable						
0–<1 portion	ref		ref		ref	
1–<2 portions	0.57 (0.39–0.84)	<0.01	0.64 (0.43–0.95)	0.03	0.57 (0.38–0.86)	<0.01
2–<3 portions	0.57 (0.39–0.84)	<0.01	0.71 (0.48–1.05)	0.09	0.64 (0.43–0.96)	0.03
3–<4 portions	0.64 (0.41–0.99)	0.05	0.75 (0.48–1.18)	0.21	0.70 (0.44–1.11)	0.13
4–9 portions	0.52 (0.31–0.87)	0.01	0.62 (0.37–1.06)	0.08	0.53 (0.30–0.93)	0.03

Model 1: Adjusted for age and gender. Model 2: Adjusted for age, gender, smoking, alcohol, wealth, education, and living alone. Model 3: Adjusted for age, gender, smoking, alcohol, wealth, education, living alone, cognition, depressed mood, diabetes, and hyperlipidemia. CI: confidence interval, ref: reference.

## Supplementary Materials

The following are available online at https://www.mdpi.com/2072-6643/12/12/3882/s1, Figure S1: Self-completion questionnaires for fruit and vegetable consumption at English Longitudinal Study of Ageing wave 4 and conversion rates into portion.
