# Supplementary material for: Fruit and Vegetable Consumption and Incident Prefrailty and Frailty in Community-Dwelling Older People: The English Longitudinal Study of Ageing
Source: Nutrients. 2020 Dec 18;12(12):3882. doi: 10.3390/nu12123882 (PMC7766547; doi:10.3390/nu12123882)
Supplement: Supplementary file 1 [file nutrients-12-03882-s001.pdf]

35 **Using the measures below, how much of the following did you eat yesterday?**
Please read through the whole list before answering.
For each food type, write '0' if none eaten.

	Write in number	
Salad (cereal bowlfuls)		x 1
Tablespoons of vegetables (raw, cooked, frozen or tinned) *Include peas and greens. Do not include potatoes*		x 1/3
Tablespoons of pulses such as baked beans, red kidney beans, lentils		x 1/3 (up to 1)
Tablespoons of other dishes mainly made from vegetables or pulses, such as vegetable lasagne or vegetable curry		x 1/3

36 **Using the measures below, how much of the following did you eat yesterday?**
Please read through the whole list before answering.
For each food type, write '0' if none eaten.

	Write in number	
Average handfuls of very small fruit, such as grapes, berries		x 1/2
Small fruit, such as plums, satsumas		x 1/2
Medium fruit, such as apples, bananas, oranges		x 1
Half a large fruit, such as grapefruit		x 1
Average slices of a very large fruit, such as melon		x 1
Tablespoons of frozen or tinned fruit		x 1/3
Tablespoons of dried fruit, such as raisins, apricots		x 1 (up to 1)
Tablespoons of other dishes made mainly from fruit such as fruit salad or fruit pies		x 1/3
Small glasses of fruit juice		x 1 (up to 1)

Figure S1. Self-completion questionnaires for fruit and vegetable consumption at English Longitudinal Study of Ageing wave 4 and conversion rates into portion.
